# Development of the Binocular Circuit

**DOI:** 10.1146/annurev-neuro-111020-093230

**Published:** 2024-07-01

**Authors:** Eloísa Herrera, Alain Chédotal, Carol Mason

**Affiliations:** 1 Instituto de Neurociencias (CSIC-UMH), Consejo Superior de Investigaciones Científicas and Universidad Miguel Hernández, Alicante, Spain; 2 Institut de la Vision, INSERM, Sorbonne Université, Paris, France; 3 Institut de Pathologie, Groupe Hospitalier Est, Hospices Civils de Lyon, Lyon, France; 4 Université Claude Bernard Lyon 1, MeLiS, CNRS UMR5284, INSERM U1314, Lyon, France; 5 Departments of Pathology and Cell Biology, Neuroscience, and Ophthalmology, Zuckerman Institute, Columbia University, New York, NY, USA

**Keywords:** retinal ganglion cell, optic chiasm, transcriptomics, ciliary margin, axon targeting, evolution

## Abstract

Seeing in three dimensions is a major property of the visual system in mammals. The circuit underlying this property begins in the retina, from which retinal ganglion cells (RGCs) extend to the same or opposite side of the brain. RGC axons decussate to form the optic chiasm, then grow to targets in the thalamus and midbrain, where they synapse with neurons that project to the visual cortex. Here we review the cellular and molecular mechanisms of RGC axonal growth cone guidance across or away from the midline via receptors to cues in the midline environment. We present new views on the specification of ipsi- and contralateral RGC subpopulations and factors implementing their organization in the optic tract and termination in subregions of their targets. Lastly, we describe the functional and behavioral aspects of binocular vision, focusing on the mouse, and discuss recent discoveries in the evolution of the binocular circuit.

## BASIC ANATOMY OF THE BINOCULAR CIRCUIT

The binocular circuit comprises the neural network that integrates information perceived in each eye to create a coherent three-dimensional (3D) percept of the visual world. In order for this network to function properly, the two eyes must align so that their visual fields overlap, and the brain must be able to accurately match or resolve the mismatch of the corresponding points in the images received by each eye.

The binocular circuit forms in all vertebrate species through a complex and fascinating process spanning multiple stages of embryonic and perinatal development. A key event during the formation of this circuit is the differentiation and maturation of retinal ganglion cells (RGCs), whose axons project to the brain. These cells are born in the innermost layer of the developing retina and migrate radially to form the ganglion cell layer. Once differentiated, RGCs extend their axons to exit the retina through the optic disc and enter the optic stalk, which becomes the optic nerve, and reach the brain at the developing ventral diencephalon. There, RGC axons form the optic chiasm, the structure in which some visual axons from each eye cross the midline to the opposite brain hemisphere (contralateral axons), while others remain on the same side (ipsilateral axons). In mammals, contralateral axons originate from the nasal side of the retina (closer to the nose), while ipsilateral axons originate from the temporal side of the retina (closer to the temple). This partial decussation at the chiasm allows visual stimuli from both eyes to integrate properly in the brain to form a complete 3D image of the environment.

After leaving the optic chiasm, RGCs continue in their path to project to the dorsolateral geniculate nucleus (dLGN) of the thalamus and the superior colliculus (SC), which are essential for visual processing. The dLGN relays visual information to the visual cortex (V1), located in the occipital lobe of the brain. The integration and convergence of sensory information from both eyes to binocularly driven neurons in the cortex constitutes the foundation for stereopsis (depth perception) and conscious visual perception, while the SC directs visually guided behaviors (Stryker & Schiller 1975). In contrast, non-image-forming nuclei such as the olivary pretectal nucleus and the suprachiasmatic nucleus mediate the pupillary reflex, saccadic movements, image stabilization, and entrainment of the circadian clock, hormone rhythms, and sleep cycles.

Here, we review recent breakthroughs in our understanding of the mechanisms underlying the formation of the binocular circuit and its molecular designation, processing of visual signals in the binocular circuit, and evolutionary considerations.

## MECHANISMS UNDERLYING AXONAL DIVERGENCE AT THE OPTIC CHIASM

The midline of the ventral diencephalon is rich in guidance cues, and during development, visual axons must sense and appropriately integrate multiple signals to ensure accurate wiring of the binocular circuit. The divergence of ipsi- and contralateral axons at the midline, a classic model for axon guidance, relies on the interaction between cell-surface receptors on RGC growth cones and their corresponding ligands in the cellular milieu as well as timed orchestration of intracellular signaling events. The intracellular signaling pathways activated by receptor-ligand interactions then direct the remodeling of the cytoskeleton, ultimately inducing either repulsive or attractive responses in the growth cone (Sánchez-Huertas & Herrera 2021).

### Signaling Pathways Implementing Decussation of Visual Axons at the Chiasm

In mice, the first optic axons entering the chiasmatic area arise from the dorsocentral retina. These pioneer axons enter a radial glial cell palisade in the prospective chiasm region at embryonic day (E)12 and course along a boundary defined by a transient neuronal population expressing cell-surface proteins such as stage-specific embryonic antigen-1 (SSEA-1), cluster of differentiation 44 (CD44), beta-tubulin, and chondroitin sulfate proteoglycans to cross to the other side of the developing brain. Some pioneer axons do not enter the palisade and instead turn directly into the ipsilateral optic tract. It has been suggested that these pioneer axons guide follower axons as they exit the eye, along the midline, and through the optic tract (Soares & Mason 2015). What guides pioneer axons and whether they become functional later or solely aid in guiding subsequent permanent axons through trans-axonal signaling remain unclear, but the fact that the pioneer ipsilateral axons completely vanish after the refinement process (Soares & Mason 2015) supports the latter hypothesis.

The advent of new tracers in combination with live imaging brought the discovery of how and where ipsi- and contralateral axonal growth cones decussate and led to an assignment of molecules that direct decussation. The guidance molecules Slits, Semaphorin 6D, and vascular endothelial growth factor A (VEGF-A) are all expressed at the midline on resident neurons and glia, and together with their receptors expressed by RGC axons, they implement the proper formation and shape of the contralateral pathway (Erskine et al. 2011, Kuwajima et al. 2012, Plump et al. 2002). Contralateral axons also secrete Sonic Hedgehog (Shh), which is thought to repel ipsilateral axons that express the Shh receptor Boc (Peng et al. 2018). While Shh could mediate ipsi-/contralateral RGC axon defasciculation and divergence as axons reach the chiasm, it is unclear why ipsilateral axons respond to Shh only at the chiasm and not along the entire pathway. A newly defined signal for implementing crossing at the ventral diencephalic midline is the CXC motif chemokine ligand 12 [CXCL12; also known as stromal cell–derived factor 1 (SDF-1)], expressed by the meninges bordering the optic pathway, and its receptor CXCR4, expressed in both ipsi- and contralaterally projecting RGCs (Le et al. 2024). Knockouts of CXCL12 display an increased ipsilateral projection, implicating the meninges as an added component of axon guidance at the chiasm midline.

**Figure 1:**
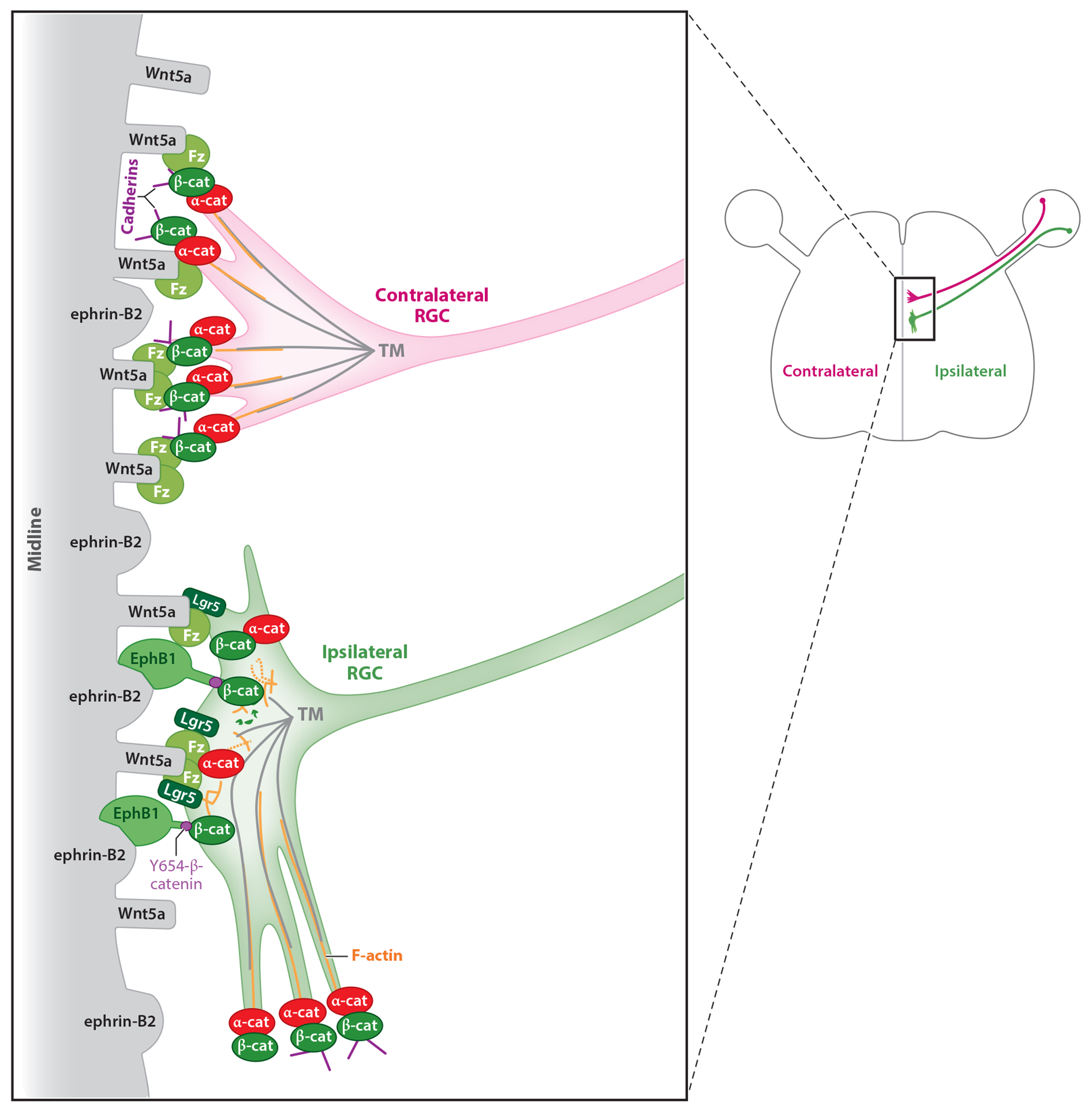
Wnt5a-dependent regulation of RGC axonal divergence at the chiasm midline. As RGC axons reach the midline, they encounter Wnt5a. All RGC axons express Wnt receptors (i.e., Fz). Upon binding to Wnt5a, Fz receptors trigger local accumulation of β-catenin, a key protein that connects α-catenin, cadherins, and actin filaments. The formation and stabilization of catenin/cadherin complexes promote the polymerization of actin filaments and implement forward growth of the axon, for contralateral RGCs (*top*). In ipsilateral RGCs (*bottom*), the presence of the tyrosine kinase receptor EphB1, expressed only in ipsilateral RGCs, comes into play: Upon binding to ephrin-B2, also expressed by midline cells, EphB1 can locally phosphorylate β-catenin at Y654 (Y654-β-catenin, *purple circles*). The phosphorylated form of β-catenin has reduced affinity for cadherins, hampering the formation of cadherin/actin complexes that produce forward growth. The nonphosphorylated excess β-catenin promotes cytoskeleton stabilization in the aspect of the growth cone interacting with midline cells and stimulates axon steering away from the midline. In addition to the shared Wnt receptors expressed by both RGC subpopulations, ipsilateral RGCs also express other Wnt receptors, including Lgr5, Fz1, and Fz8, which may also contribute to the act of growth cone turning. Abbreviations: EphB1, ephrin type B receptor 1; Fz, frizzled; Lgr5, leucine-rich repeat-containing G protein–coupled receptor 5; MT, microtubule; RGC, retinal ganglion cell.

The tyrosine kinase receptor ephrin type B receptor 1 (EphB1) is highly enriched in ipsilateral RGCs and by interacting with its cognate ligand ephrin-B2, expressed on midline radial glia, mediates repulsion of ipsilateral RGC axons away from the midline (Williams et al. 2003). Very recently, the Wingless (Wnt) pathway was discovered to function in conjunction with EphB1/ephrin-B2 repulsive signaling (Morenilla-Palao et al. 2020) and provides a more comprehensive molecular picture of the guidance of ipsilateral axons (Figure 1). Like ephrin-B2, Wnt5a is highly expressed at the midline, but the exposure of contralateral and ipsilateral axons to Wnt5a triggers opposing responses: Contralateral axons exhibit enhanced growth, and ipsilateral axons halt their growth. Canonical Wnt signaling is activated when frizzled (Fz) receptors bind to Wnt, as in many settings, inducing the accumulation of β-catenin and its translocation to the nucleus to activate transcription. However, in differentiated RGCs, canonical Wnt signaling is not activated. Instead, accumulation of β-catenin stimulates cadherin-catenin complexes to link to the actin cytoskeleton, stabilizing actin filaments and enabling forward extension of contralateral RGC axons (Figure 1). In ipsilateral axons, the activation of EphB1 by ephrin-B2 phosphorylates β-catenin (Morenilla-Palao et al. 2020), likely preventing that formation of a cadherin/actin complex, facilitating growth cone turning (Kim & Lee 2001) (Figure 1). While the exact role of adenomatous polyposis coli protein 2 (Apc2) in midline axon guidance remains uncertain, it is noteworthy that this protein, known for its involvement in regulating microtubule dynamics (Arbeille & Bashaw 2018, Mattie et al. 2010), exhibits reduced expression levels in ipsilateral RGC growth cones compared to their contralateral counterparts (Morenilla-Palao et al. 2020). The secreted frizzled-related proteins Sfrp1 and Sfrp2, antagonists of Wnts, are also expressed in RGC axons, and in their absence, axons become loosely defasciculated at the optic chiasm (Marcos et al. 2015), but it is not clear whether these Wnt antagonists differentially affect ipsi- versus contralateral axons. Further experiments are needed to fully understand how the noncanonical Wnt pathway promotes a positive or a negative response in contralateral and ipsilateral RGC axons at the chiasm.

### Transcriptional Control of Axonal Trajectories

The expression of guidance receptors and intracellular signaling pathways that direct the ipsi- versus contralateral axonal trajectories is tightly orchestrated by regulatory transcriptional mechanisms. The zinc finger protein of the cerebellum 2 transcription factor (Zic2), transiently expressed in ipsilateral but not contralateral RGCs, regulates axon midline avoidance (Escalante et al. 2013, Herrera et al. 2003) by controlling the expression of EphB1 (Herrera & Garcia-Frigola 2008) as well as members of the Wnt signaling pathway, including *Fzd1, Fzd8*, Apc2, and the leucine-rich repeat-containing G protein–coupled receptor 5 (Lgr5) (Morenilla-Palao et al. 2020). Thus, *Zic2* stands as a master transcription factor orchestrating the ipsilateral pathway.

The LIM (LIN-11, Isl-1 and MEC-3) homeodomain (LIM-HD) transcription factor Isl2 was initially proposed as a determinant of guidance for contralaterally projecting neurons (Pak et al. 2004). Even though Isl2 is excluded from ipsilateral RGCs, it is expressed in only 40% of contralaterally projecting RGCs, and later studies confirmed that Isl2 itself does not determine contralateral trajectories but is related to non-ON-OFF direction-selective RGC fate (Kay & Triplett 2017). The SoxC family of transcription factors, particularly *Sox4, 11*, and *12*, are key for the differentiation of contralateral RGCs by inhibiting Hes family basic helix-loop-helix transcription factor 5 (*Hes5*) expression. SoxC genes upregulate the expression of Plexin-A1 and Nr-CAM, which, as mentioned above, guide contralateral RGC axons across the midline (Kuwajima et al. 2012). These transcription factors also seem to repress ipsilateral RGC differentiation, as mice lacking SoxCs have more ipsilateral projections (Kuwajima et al. 2017).

Formerly, a blend of candidate approaches and serendipitous discoveries illuminated this rather small number of transcription factor families associated with ipsi- or contralateral cell fate for chiasm decussation. In recent years, significant strides have been made in identifying additional transcriptional control of cell fate through breakthroughs in genome-wide technologies. In particular, the assay for transposase-accessible chromatin coupled to deep sequencing (ATAC-seq) has provided new avenues for unbiased exploration of transcriptional regulation. ATAC-seq profiles of ipsilateral and contralateral RGCs defined around 150,000 accessible regions in the chromatin of RGCs at the time that axons navigate the optic chiasm region. The comparison of differentially accessible regions in ipsi- and contralateral RGCs revealed that axonal trajectory selection is highly influenced by the differential occupation of enhancers rather than promoters (Fernández-Nogales et al. 2022).

Footprint analysis of the ATAC-seq data combined with RNA sequencing (RNA-seq) data sets confirmed the transcription factors previously identified in defining RGC axon laterality such as *Zic2* and *SoxCs*. When combined with chromatin immunoprecipitation sequencing (ChIP-seq) assays, these analyses newly demonstrated that Zic2 binds to the regulatory regions of many receptors previously implicated in axon pathfinding at the midline in the visual and other systems such as bone morphogenetic protein receptor type 1 (Bmpr1) or uncoordinated 5 type c receptor (Unc5c) (Fernández-Nogales et al. 2022). Interestingly, Zic2 appears to function as an inducer for Bmpr1b but a repressor for Unc5c. This finding makes sense because axons expressing this Netrin receptor cross the midline and project to the opposite retina to generate a transient retino-retinal (R-R) connection during perinatal stages that may be important for the synchronization of retinal waves (Murcia-Belmonte et al. 2019). Zic2 also binds to accessible regulatory regions of the Neuropilin2 gene (*Nrp2*), which is also specifically expressed in ipsilateral RGCs (Fernández-Nogales et al. 2022).

An unanticipated finding is that integrins, including Itga5, are differentially expressed in ipsilateral RGCs and most of them display Zic2 binding in their genomic sequences (Fernández-Nogales et al. 2022). Integrins have been recently implicated in ipsilateral RGC targeting in combination with the extracellular matrix (ECM) protein nephronectin (Su et al. 2021, Tsai et al. 2022). In combination with the serotonin transporter (SERT), integrins make ipsilateral RGCs more vulnerable to injury, and if both are deleted, ipsilateral RGCs regenerate better (Kingston et al. 2021). These results implicate a prominent function for integrins in ipsilateral RGCs, but further work is needed to understand their role.

The multiomic screen also highlighted potential candidates for contralaterality. The POU class 4 homeobox 1 (Pou4f1) and POU class 4 homeobox 2 (Pou4f2) have footprints in more than 30% of the genes that are differentially expressed in contralateral RGCs, in line with previous findings that Pou4f1 and Pou4f2 are expressed in contralateral RGCs (Quina 2005). Downregulation of Pou4f1 in contralateral RGCs during embryonic stages results in pronounced axon stalling at the midline (Fernández-Nogales et al. 2022). Gene ontology analysis of the genes potentially regulated by Pou4f1 and Pou4f2 retrieved terms associated with semaphorin-mediated axon guidance, implicating these factors in controlling signaling pathways mediated by semaphorins, as discussed above, as essential for midline crossing (Kuwajima et al. 2012). Pou4f1 also surprisingly influences the expression of γ-synuclein, which is specifically expressed in contralateral RGCs and also essential for crossing (Fernández-Nogales et al. 2022).

**Figure 2:**
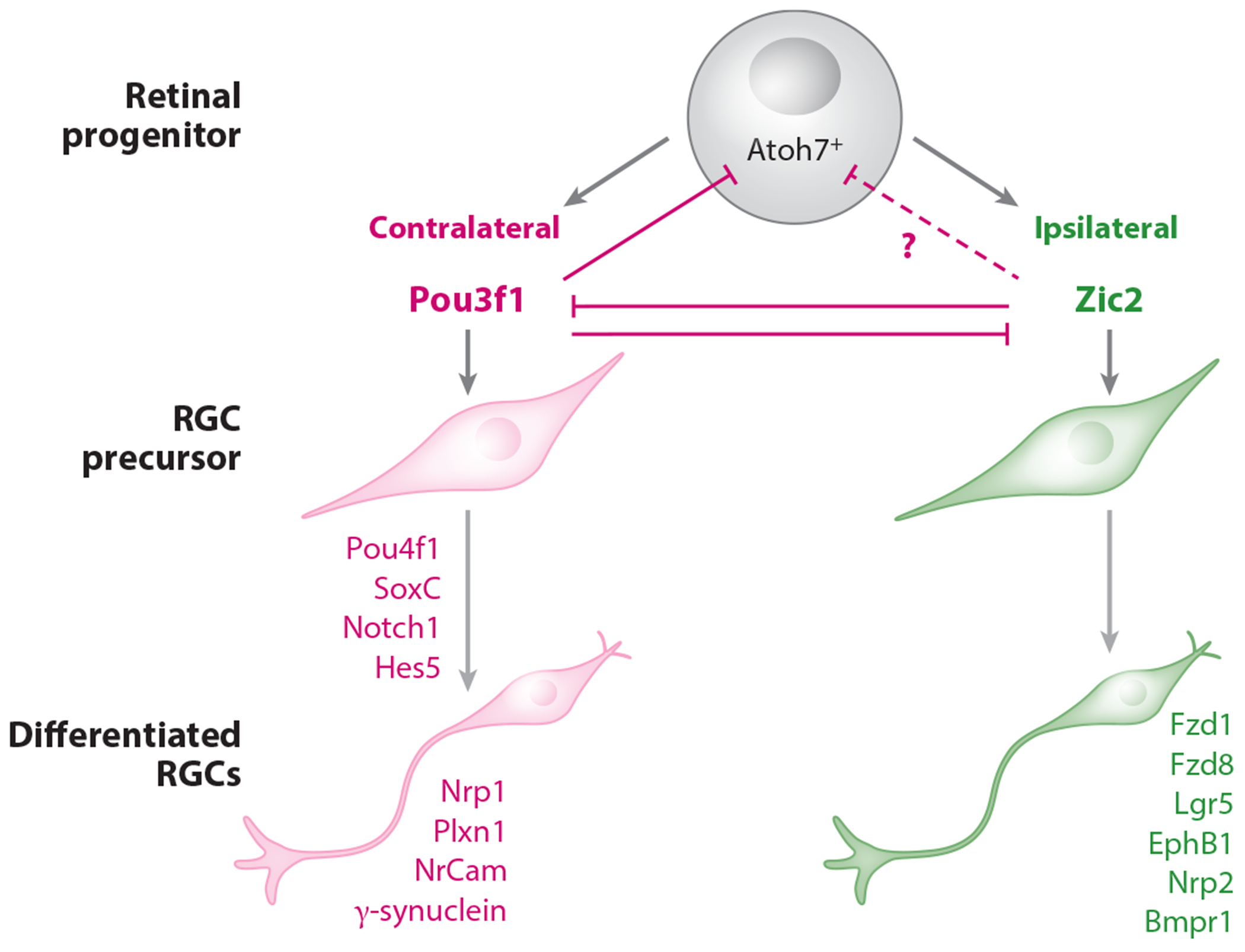
Regulatory transcriptional networks underlying contralateral/ipsilateral RGC identity. All retinal neuronal progenitors express Atoh7 before differentiating into RGCs. Although the trigger is not known, some progenitors begin to express Pou3f1, which then represses the expression of Atoh7 and promotes neuronal differentiation. The expression of Pou3f1 induces the contralateral gene program, including Pou4f1 and SoxCs, which in turn controls the expression of Notch1 and Hes5. In contrast, recently differentiated ipsilateral neurons express Zic2, which may repress Pou3f1 and possibly Pou4f1, inhibiting the contralateral program and stimulating the expression of a series of receptors (EphB1, Fzd1, Fzd8, Lgr5) that promote repulsion from the midline. It is not clear whether Zic2 also represses Atoh7 directly or via Pou3f1 to ensure ipsilateral identity. The Lhx family (not shown) may also play a role in regulating the ipsilateral pathway by modulating the expression of Zic2 targets involved in Wnt signaling, although it is unclear whether these transcription factors are switched on by Zic2 or act as independent cofactors. Abbreviations: Atoh7, atonal BHLH transcription factor 7; Bmpr1, bone morphogenetic protein receptor type 1; EphB1, Eph receptor B1; Fz, frizzled; Hes5, Hes family BHLH transcription factor 5; Lgr5, leucine-rich repeat-containing G protein–coupled receptor 5; Lhx, LIM homeobox transcription factor; Notch1, Notch receptor 1; NrCam, neuronal cell adhesion molecule; Nrp1, neuropilin 1; Pou3f1, POU class 3 homeobox 1; Plxn1, plexin A1; RGC, retinal ganglion cell; Zic2, zinc finger protein of the cerebellum 2.

Another member of the Pou family, POU class 3 homeobox 1 (Pou3f1), also retrieved in the footprint screen discussed above (Fernández-Nogales et al. 2022), is essential for ensuring contralateral fate. Transcriptomic characterization of Pou3f1-positive RGCs together with Pou3f1 functional analyses revealed that this factor upregulates the expression of contralateral markers, including Pou4f1, and suggested that Pou3f1 represses Zic2 (Fries et al. 2023). In support of these data, loss of Pou3f1 results in an expanded domain of the Zic2-positive cell population, while overexpression of Pou3f1 leads to a reduced number of Zic2-positive cells. Pou3f1 binds to the Zic2 promoter (Fries et al. 2023). Conversely, Zic2 overexpression leads to a reduction in Pou3f1 levels (Morenilla-Palao et al. 2020). Remarkably, Pou3f1 is expressed in postmitotic atonal homolog BHLH transcription factor 7 (Atoh7)-negative cells, and overexpression of Pou3f1 in postnatal retina long after Atoh7 is downregulated is sufficient to promote the generation of RGCs. Together, these findings put Pou3f1 in the spotlight as playing a critical role in controlling contralateral RGC production, independent of Atoh7 (Figure 2).

In summary, the advent of cutting-edge ’omics technologies has substantially contributed to the unbiased identification of critical differences between gene expression, chromatin accessibility, and transcription factor binding in ipsilateral and contralateral RGCs and has retrieved new genes and mechanisms implicated in regulating the divergence of RGC fate and trajectory at the chiasm midline. Despite these advances, the facts that Zic2 does not totally switch the laterality of all electroporated RGCs (Herrera & Garcia-Frigola 2008) and that most RGCs still project contralaterally in the Pou3f1 mutants (Fries et al. 2023) suggest a much more complex scenario in the control of axonal laterality. That is, there must be more than one simple master gene for contra- or ipsilateral projection fate.

### Neurogenesis and Ipsi-/Contralateral RGC Fate

A still-unresolved question for all neural systems is, What controls particular transcription factor expression at a given time or in a given sequence to determine specific cell fate outcomes? Does the timing of neurogenesis impact specific transcription factor expression as a prelude to RGC subtype identity? This question is especially germane for the expression of Pou3f1 and Zic2 (Figure 2). Pou3f1 is reported to turn off Atoh7 toward contralateral RGC differentiation, but in precursors giving rise to ipsilateral RGCs, Atoh7 expression lingers as Zic2 is expressed and then is later turned off (Wang et al. 2016). Moreover, given the extended timeline for contralateral RGC production (E11–18) versus the more brief neurogenesis of ipsilateral RGCs in the middle of this period (E14–18), when do Zic2 and Pou3f1 mutually repress one another? Recent studies using single-cell RNA-seq to profile RGCs from embryonic and postnatal animals have suggested that subtype diversification arises as a gradual, but asynchronous, fate restriction of postmitotic multipotential precursors and that some types of retinal neurons are not identifiable until some time after their differentiation (Giudice et al. 2019, Rheaume et al. 2018, Shekhar et al. 2022). Axonal laterality appears to be specified before cell type identity is completely set, and immature RGCs are likely multipotential even after selecting the ipsi- or contralateral trajectory, an idea also supported by recent work demonstrating that there is an intermediate stage between postmitotic and full differentiation where cells still can be reprogrammed (Fries et al. 2023).

At E10–12, the retinal pigment epithelium (RPE) and neural retina differentiate as the eyecup invaginates, and at the hinge of the invagination, the ciliary marginal zone (CMZ) arises and remains as the most peripheral part of the developing retina. This zone is a site of ongoing RGC neurogenesis during the lifetime of lower vertebrates (Fernández-Nogales et al. 2019, Wan et al. 2016) and is now accepted as a site for RGC neurogenesis in the embryonic mouse (Bélanger et al. 2017, Marcucci et al. 2016). Ipsilateral RGCs are born in a foreshortened period in the middle of contralateral RGC generation (Marcucci et al. 2019), and a significant percentage of ipsilateral RGCs arise from the CMZ (Marcucci et al. 2016, Slavi et al. 2023). Growth factors such as insulin-like growth factor binding protein 5 (IGFbp5) and insulin-like growth factor 1 (IGF1) expressed in the early ventrotemporal segment and in non-ventrotemporal RGCs, respectively (Wang et al. 2016), are candidates for ipsi- versus contralateral RGC neurogenesis and specification.

The cell cycle regulator cyclin D2 has emerged as an orchestrator of ipsilateral RGC neurogenesis in the CMZ. Cyclin D2 is strongly expressed in a population of CMZ neural progenitors and regulates the duration of the cell cycle to yield the proper proportion of ipsilateral RGCs (Iwai-Takekoshi et al. 2018; Marcucci et al. 2016, 2019; Slavi et al. 2023). In the albino retina, proven to be a useful tool to study the CMZ as related to neurogenesis, the timing of ipsilateral RGC neurogenesis is disrupted (Bhansali et al. 2014, Rachel et al. 2002, Slavi et al. 2023). Cyclin D2 is expressed at lower levels in the albino CMZ during ipsilateral RGC neurogenesis, and if cyclin D2 is conditionally removed in the CMZ of pigmented mice, or overexpressed in albino mice by introduction of an L-type calcium channel agonist, the ipsilateral RGC number is decreased or rescued, respectively (Slavi et al. 2023).

The absence or perturbation of melanin in the RPE of albino individuals is linked to a reduced production of ipsilateral RGCs (Bakker et al. 2022), but it is unclear to what extent the RPE and factors associated with melanin production govern RGC neurogenesis to influence cell fate. Recent advances have illuminated the morphogens and transcriptional programs accountable for CMZ development, which are potentially critical for the determination of ipsi- and contralateral RGC populations. The differentiation of the CMZ versus the neural retina and RPE in their proper spatial proportions during early eyecup development results from a delicate equilibrium of Wnt, fibroblast growth factor (FGF), and other signaling pathways (Balasubramanian et al. 2021, Buono et al. 2021). Additionally, junctions exist between RPE and RGC progenitors, and through calcium dynamics, ATP-purinergic signaling influences neurogenesis (Pearson et al. 2005). RPE–neural retinal cell junctions, both adherens and gap junctions, are improperly arrayed in the albino embryonic eye (Iwai-Takekoshi et al. 2016). Therefore, it is possible that in the albino, perturbed junctions impair the release of molecules from the RPE driving RGC progenitor neurogenesis or allow the release of inflammatory/deleterious molecules that arise in the absence of the pigment pathway, in either case leading to an imbalanced output of ipsi- and contralateral RGCs.

## FROM THE OPTIC CHIASM TO TARGETS IN THE BRAIN

After RGC axons have decussated to ipsi- and contralateral sides of the brain, they travel along the outer limits of the diencephalon to form the optic tract and then extend toward their image-forming targets in the thalamus, midbrain, and a number of other targets of non-image-forming RGCs. After RGC axons enter the image-forming nuclei, they establish a rough Eph-ephrin-mediated topographic map. RGC terminals exuberantly branch and undergo a process of refinement or pruning to connect to their target neurons with a proper balance of synapses. The refinement process establishes the final retinotopic map as well as eye-specific segregation of ipsi- and contralateral terminals in the thalamus that then transmit information to the visual cortex, forming ocular dominance columns or maps in higher mammals. Both topographic refinement and ipsi-/contralateral segregation are mediated by neural activity that is spontaneously generated in the developing retina during perinatal stages (Arroyo & Feller 2016, Huberman et al. 2008).

Compared with the molecular directives for guidance at the optic chiasm decision point, much less is understood about the molecular underpinnings of guidance to and within targets. We next review what is known about ipsi- and contralateral-specific RGC axon arrangements in the optic tract and the molecular mechanisms of target entry and termination in specific target subzones.

### Pretarget RGC Axon Organization in the Optic Tract

In *Xenopus* and zebrafish, RGC axon sorting in the optic tract involves glycoproteins that stimulate contact-triggered fasciculation and repulsive responses between dorsal and ventral axons for cohesive and topographically correct tract formation (Cioni et al. 2018, Hörnberg et al. 2016, Spead et al. 2023). In the mouse, as they exit the chiasm, ipsi- and contralateral RGC axons segregate and fasciculate with their own subtype (Sitko et al. 2018). In vitro, ipsilateral axons (from ventrotemporal retina) have a greater affinity with one another than with contralateral axons. The molecules implementing sorting and affinities of ipsilateral versus contralateral axons, and the potential contributions of glial cells to axon segregation in the optic tract (Lee et al. 2019), are not well understood. Dystroglycan is important for general coherence of the optic tract, and if mutated, axons form small patches in the dLGN and tectum (Clements & Wright 2018). Other glycoproteins such as glypicans might shape optic tract fasciculation patterns as it does in lower vertebrates, since it is more highly expressed in ipsilateral RGCs (Wang et al. 2016).

### Entry into Target Regions and Targeting Eye-Specific Subzones in the Visual Nuclei

Are there molecular factors implementing entry of the appropriate brain region once axons reach the bounds of their target? In amphibians, morphogens and guidance factors such as FGF-2 and Netrin/Slit mediate the avoidance of areas ventral and medial to the optic tectum and implement tectal entry (e.g., see McFarlane et al. 1995). In the mouse, the molecular mechanisms regulating the entry of RGC axons into specific image-forming target nuclei are poorly characterized (Seabrook et al. 2017, Tuttle et al. 1998). Molecules implicated in axon targeting into specific non-image-forming centers include cadherin-6 for targeting the olivary pretectal nuclei (Osterhout et al. 2011) and contactin-4 (CNTN4) and amyloid precursor protein (APP) for targeting the nucleus of the optic tract (Osterhout et al. 2015). Likewise, the transmembrane semaphorin Sema6A and its binding partners Plexin-A2/A4 control the targeting of some direction-sensitive RGCs to the medial terminal nucleus (MTN) (Sun et al. 2015). Interestingly, in this system, Sema6A acts as a receptor and Plexin-A2/A4 as ligands. Abnormal innervation of the MTN occurs after conditional ablation of the Netrin1 receptor deleted in colorectal cancer (DCC) in RGCs (Vigouroux et al. 2020).

To innervate the dLGN, a subset of RGC axons terminating in the SC emit a collateral to the dLGN by postnatal day (P)0. Retinal axons begin to reach, enter, and branch within the dLGN around E16 (Godement et al. 1984). Axons must defasciculate to enter target regions. Semaphorin 6D and Plexin-A1, both expressed in RGCs and the dLGN, interact to ensure coherent organization and defasciculation prior to target entry of retinogeniculate axons within the optic tract at the outer limits of the dLGN and proper target invasion (Prieur et al. 2023).

Inside the retinorecipient nuclei, except for the suprachiasmatic nuclei, the right and left eye inputs are segregated, and axons terminate in a map that is topographically related to retinal inputs and in eye-specific domains in stereotypic locations in the targets (Beier et al. 2021, Huberman et al. 2008). In the mouse, retinal axon collaterals from both eyes that innervate the dLGN are intermingled at P3. From P4, retinal axon terminals begin to branch profusely, and concomitantly exuberant branches are pruned (Dhande et al. 2011), leading to the segregation of ipsi- and contralateral axons in specific regions of the dLGN by P7 (Godement et al. 1984, Hong et al. 2019, Jaubert-Miazza et al. 2005). Interrogation of the molecular underpinnings of axon-target matching and subtarget innervation, especially regarding eye-specific inputs, has again pointed to glycoprotein and adhesion molecules. The transmembrane protein Ten_m3, a member of the teneurin (Ten-m/Odz) family, is expressed in a gradient in the developing visual pathway and is highest in regions that include the ipsilateral recipient territories in the dLGN, and mice lacking Ten_m3 show abnormalities in the mapping of ipsilateral projections (Leamey et al. 2007). Mutation of Ten_m4, expressed uniformly in the retina, leads to an increase both in EphB1 expression and in the number of ipsilateral RGCs (Young et al. 2023). Ten_ms are glycoproteins that appear to act homophilically between axon and target cells, but whether such homophilic adhesion is a prelude to synapse formation, as proposed for the adhesion molecule Down syndrome cell adhesion molecule (DSCAM) in both tract cohesion and synaptogenesis in targets (Santos et al. 2018), needs further investigation.

The extracellular glycoproteins neural epidermal growth factor-like-like 2 (Nell2) and nephronectin (NPNT) represent more specific ipsilateral RGC targeting factors for the mouse dLGN and the SC (Nakamoto et al. 2019, Su et al. 2021). Nell2 is expressed in the dorsomedial region of the dLGN, where ipsilateral RGC axons terminate and which contralateral axons avoid. In vitro, Nell2 repels contralateral, but not ipsilateral, RGC axons and appears to act as an inhibitory guidance molecule preventing contralateral axons from entering the ipsilateral dLGN territory. NPNT is expressed by ipsilateral RGCs and is required for the targeting of ipsilateral axons to the deep sublamina of the SC through a cell-ECM recognition mechanism.

### Activity-Dependent Remodeling of Visual Terminals in Targets

Neural activity has been invoked as an important force shaping binocular projections, working in concert with glial cell activity in pruning excess arbors and synapses during the developmental refinement of eye-specific inputs (Goodman & Shatz 1993, Schafer et al. 2012). Little is known about how ipsi- and contralateral RGCs are differentially affected by spontaneous retinal activity. Interestingly, if cells fated to be ipsi- or contralateral aberrantly project to visual nuclei on the inappropriate side, as in the EphB1 knockout and the albino, RGC axons still target correctly but form a segregated patch of terminals in or near the target. The segregation of these patches is activity dependent (Rebsam et al. 2009, 2012).

Interestingly, when glutamate release is specifically blocked in ipsilateral RGCs, ipsilateral targeting is normal, but contralateral RGCs do not refine and continue to innervate the ipsilateral territory in the adult mice (Koch et al. 2011). One of the few molecules known to be implicated in mediating activity-dependent innervation by ipsilateral RGCs is SERT, which is expressed in ipsi- but not contralateral RGCs (Upton et al. 1999) and induced by Zic2 (García-Frigola & Herrera 2010). The loss of SERT (Salichon et al. 2001, Upton et al. 2002) or an increase of extracellular serotonin levels (Upton et al. 1999) impairs the retraction of ipsilateral fibers from the contralateral zone. It is likely that SERT clears serotonin from the zone that includes the ipsilateral target. All RGC terminals express the serotonin receptor 5-HT_1B_. The activation of 5-HT_1B_ by serotonin in contralateral terminals inhibits cyclic adenosine monophosphate (cAMP) production and calcium entry. However, ipsilateral axons expressing SERT are able to internalize serotonin, relieving 5-HT_1B_-mediated inhibition and inducing cAMP production. This produces a discrepancy in the activity levels of the afferent retinal inputs, allowing eye-specific refinement/segregation (García-Frigola & Herrera 2010, Nicol et al. 2007).

Recent computational models have predicted that the synchronization of activity-dependent retinotopy and eye-specific segregation in both brain hemispheres is essential for the proper formation and function of the circuit. This synchronization may be possible through a recently described R-R projection that interconnects both retinas during the refinement of RGC terminals at their targets and whose formation depends on the expression of the Netrin receptor Unc5c (Ackman et al. 2012, Murcia-Belmonte et al. 2019). Despite this prediction, neither the actual function of this transient R-R projection nor the necessity of activity synchronization between both retinas during perinatal stages has been experimentally confirmed.

In summary, while molecular factors underlying axon-target innervation are beginning to be identified, intrinsic retinal activity plays a crucial role in segregating ipsi- and contralateral inputs within the visual nuclei and is therefore essential to adjust the final shaping of the binocular circuit. Additional analysis is needed to identify the precise molecular mechanisms underlying retinal activity-dependent, eye-specific refinement and pruning of RGC axons.

## EVOLUTION OF THE BINOCULAR CIRCUIT

Bilateral visual projections connecting the eyes to the brain exist in all mammalian species, but the ratio of RGC axon decussation at the chiasm is extremely variable. The ipsilateral contingent is the highest in primates (about 45%) and only about 2% in mice (Herrera et al. 2003, Johnson et al. 2021, Larsson 2011). A higher ratio of ipsilateral axons in primates, together with frontal eyes and a large binocular visual field, was proposed to provide an evolutionary advantage, facilitating visuomotor control of forelimb movement, nocturnal activities, and ultimately predation (Larsson 2015).

### Conservation of RGC Midline Crossing Mechanisms

Not much is known about the conservation of RGC midline crossing mechanisms in nonrodent mammals. Transient expression of Zic2, restricted to the domain of the temporal retina hosting ipsilaterally projecting RGCs, has been reported in ferret and human embryos (Vigouroux et al. 2021). EphB1 is also confined to the temporal retina in human embryos (Lambot et al. 2005), but whether the function of laterality genes and pathways is conserved in primates is unclear. The emergence of 3D organoid and assembloid models, in which RGCs from human pluripotent stem cell–derived eye organoids extend axons into thalamic and cortical organoids (Fernando et al. 2022, Fligor et al. 2021), should help to assess visual axon crossing mechanisms in primates.

But what about lower vertebrates? Uncrossed RGC axons exist in birds, although visual connectivity has been studied only in a handful of species. In chick embryos, ipsilateral RGC projections are transient, regressing after two weeks of embryogenesis (Thanos & Bonhoeffer 1984), and Zic2 is absent from the neural retina (Herrera et al. 2003). Reptiles and amphibians, which together with mammals constitute the tetrapod superclass (four-legged vertebrates), have bilateral retinal projections. In anurans, ipsilateral axons develop only after metamorphosis, and in postmetamorphic *Xenopus, Zic2* and *EphB1* are expressed in the ventral retina domain hosting ipsilaterally projecting RGCs. Therefore, the current model suggests that tetrapods have bilateral visual projections and that Zic2 specifies ipsilateral RGC identity in the ventrotemporal retina.

**Figure 3:**
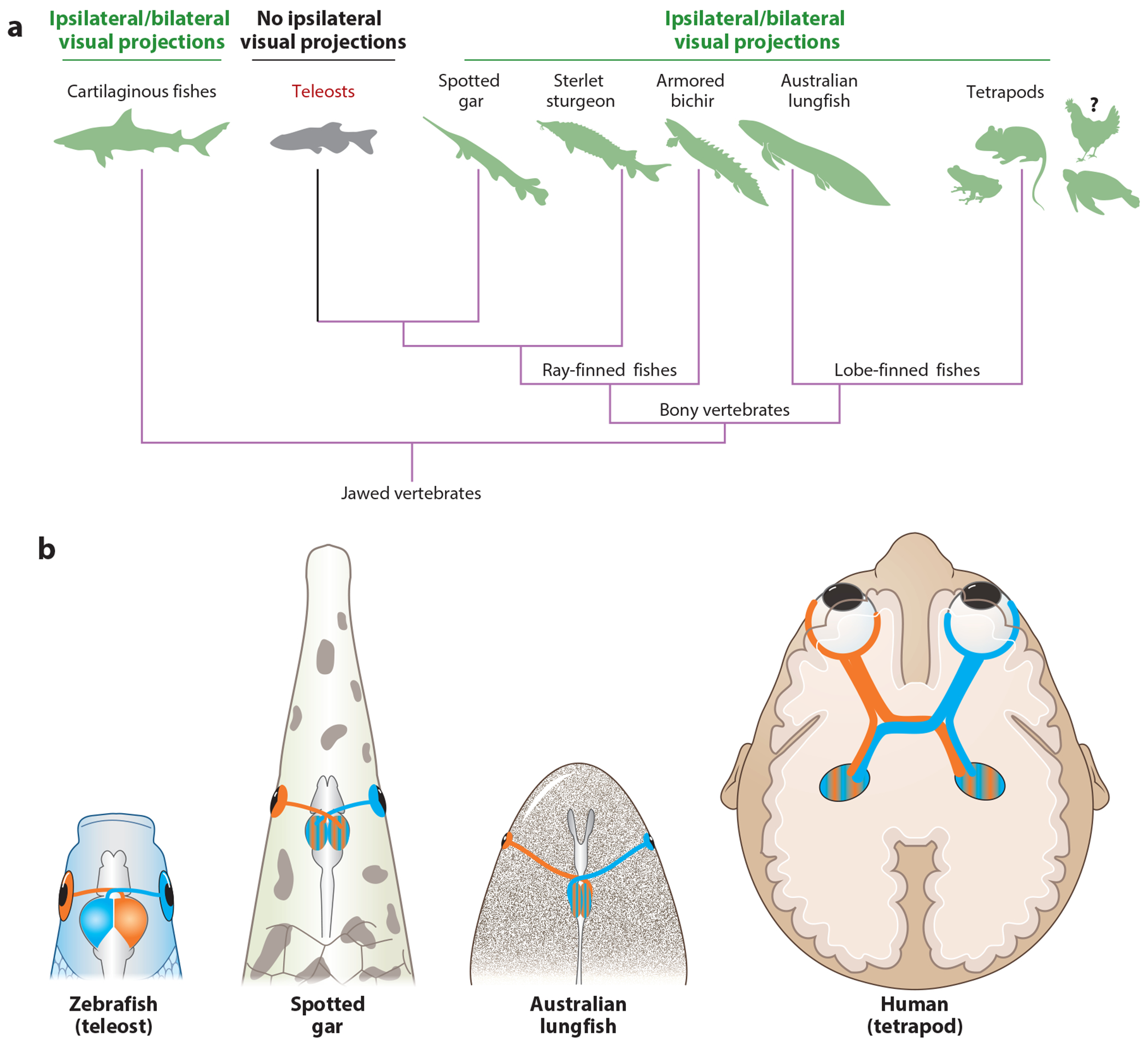
Evolution of visual projection binocularity. (*a*) Simplified phylogenetic tree of jawed vertebrates. Most teleost lineages lack ipsilateral visual projections, but eyes project bilaterally in other jawed vertebrates, including cartilaginous fishes, most basal ray-finned fishes, and lobe-finned fishes. Visual projections are also bilateral in tetrapods but have been charted only in a few bird species during development. (*b*) Diagrams of visual system connectivity in a teleost, two nonteleosts (gar and lungfish), and a tetrapod. Left and right retinal projections are shown in orange and blue, respectively. Figure adapted with permission from Vigouroux et al. (2021).

In zebrafish larvae, RGC axons fully cross the midline at the chiasm, and *Zic2* genes are not expressed in the neural retina (Vigouroux et al. 2021). This suggested that ipsilaterality emerged in tetrapod ancestors during the Late Devonian [around 360 million years ago (Mya)] at the time of the water-to-land transition. However, fishes as a group are extremely diverse, accounting for almost half of all extant vertebrates. Ipsilateral RGC projections had been reported in some fish species, albeit without any obvious phylogenetic correlation (Larsson 2013, Ward et al. 1995). Very recently, visual system laterality in fishes has been reassessed according to their phylogenetic status using high-resolution axonal tracing methods (Vigouroux et al. 2021) (Figure 3). Eleven representative bony fish species (ray-finned fishes) were selected, and seven of these were teleosts. Teleosts, by far the largest extant fish group, separated from the other extant ray-finned fish lineages about 300 Mya. Nonteleost fishes are considered more basal, although they have significantly evolved. These high-resolution tracing analyses confirmed that visual projections are exclusively contralateral in teleosts. However, they also indicated that an ipsilateral projection does in fact exist in all nonteleost basal ray-finned fishes (Vigouroux et al. 2021). This group includes a lobe-finned fish, the Australian lung fish, the closest fish relative of tetrapods. Thus, it is possible that the visual system bilaterality preceded the emergence of terrestrial vertebrates by at least 100 Mya. Ipsilateral visual projections may also exist in cartilaginous fishes and surviving jawless fishes, hagfishes, and lampreys (Kennedy & Rubinson 1977). Therefore, bilateral visual inputs might already have been present more than 500 Mya in the common ancestors of all vertebrates. Further, anatomical analysis of optic chiasm morphology in 25 fish species (Mogi et al. 2009) showed that in most teleosts, the right and left optic nerves are fully separated (with one passing above the other), precluding the possibility of interactions between RGC axons at the midline. However, this is not the case in zebrafish where optic nerves fuse and axons from the two optic nerves can interact (Hutson & Chien 2002). These studies argue that ipsilateral projections exist in teleosts but suggest that RGC laterality does not correlate with eye position, optic nerve and chiasm structure, habitat, or diet in aquatic species.

How RGC axon laterality is specified in ancient fish remains unclear as Zic2 and EphB1 are absent from the retina of the spotted gar, in which almost 5% of RGCs project to the ipsilateral tectum. Whether these uncrossed axons are transient or maintained in adult fish and whether they originate from the ventrotemporal retina as in the mouse are unknown. Likewise, the expression of Zic2 in the developing retina of other fishes with bilateral visual axons has not been studied, but its absence in the spotted gar retina suggests that the genetic program specifying RGC axon laterality could be different in nontetrapods. Remarkably, ipsilateral projections can be experimentally induced in zebrafish by overexpressing cAMP, the semaphorin receptor neuropilin1 (Dell et al. 2013), and bone morphogenetic protein 4 (BMP4) (Knickmeyer et al. 2021) or after the ectopic expression of Zic2 in RGCs (Vigouroux et al. 2021). Aberrant ipsilateral RGC projections were also observed in the zebrafish *astray* mutant [which lacks the roundabout 2 receptor (Robo2)] (Hutson & Chien 2002) and *islr2* mutant (Panza et al. 2015). Together, these findings suggest that the downstream pathways controlling ipsilateral axon guidance can still be activated in teleosts. One remaining question is the extent to which the ancient and evolutionarily conserved Wnt signaling, described above as integrating guidance receptors and influencing the remodeling of the growth cone cytoskeleton during decussation (Morenilla-Palao et al. 2020), comes into play in directing ipsilateral signaling in these primitive fish and other species.

Remarkably, a binocular circuit also exists in very primitive species, evidenced by the presence of a bona fide optic chiasm in planarians, or flatworms, some of the most simple cephalized invertebrates, that appeared much earlier than vertebrates. Planarians have two simple eyes containing one class of retinal neurons, equivalent to a photoreceptor-ganglion cell hybrid, whose axons project to visual centers. Axons from both eyes join, cross, and form an optic chiasm. Tracing studies showed that the most anterior retinal neurons project contralaterally, across the midline, and the most posterior retinal neurons project to both sides (Okamoto et al. 2005). Strikingly, Sox genes are differentially expressed in anterior and posterior photoreceptors (Lapan & Reddien 2012), and midline crossing is influenced, as in vertebrates, by Netrin and Slits (Yamamoto & Agata 2011). Together, these results strongly suggest that the bilaterality of eye connections is an ancestral feature of the visual system.

### Bilateral RGC Projections in Visual Function

Stereoscopic vision was long thought to be exclusive to higher vertebrates and particular mammals with bilateral visual inputs. To resolve depth, the brain compares two disparate retinal images, which requires the integration by visual centers of inputs coming from the right and left eyes. Thus, the existence of decussating and non-decussating RGC axons appeared as a prerequisite for 3D vision. The high fraction of ipsilateral axons in primates together with frontal eyes conferred optimal stereopsis. Accordingly, achiasmatic humans lack 3D vision. Depth perception is also severely affected in albino individuals, which have significantly reduced ipsilateral RGCs (Bakker et al. 2022, Hoffmann & Dumoulin 2015). In these conditions, the lateral geniculate nuclei are monocularly innervated, but the crossing defects do not prevent nasal and temporal RGC axons from targeting their appropriate LGN sublayers. This severely perturbs 3D vision, although other aspects of visual processing at the cortical level do not seem to be profoundly altered (Prieur & Rebsam 2017, Victor et al. 2000).

But what about stereopsis in mice, which have a very minor contingent of ipsilateral RGC axons? New functional studies demonstrating binocular vision in mice indicate the coding of visual input and eye/head position in V1 in binocular versus monocular vision (Parker et al. 2022) and natural binocular depth discrimination behavior (Boone et al. 2021). Further, the complete absence of an optic chiasm, such as in ventral anterior homeobox 1 (*Vax1*) mutant mice (Min et al. 2023), results in anomalies of depth perception, even though the ipsilateral visual cortex responds to the stimulation of the ipsilateral retina. Depth perception is also altered in a Ten_m3 knockout that displays normal RGC axon decussation but aberrant targeting of ipsilateral axons in the LGN (Leamey et al. 2007). However, in both cases, visual deficits could be, at least in part, non-cell-autonomous as mutations were not restricted to RGCs. Conclusive evidence supporting a key role for ipsilateral RGCs in stereopsis and prey capture in mice was obtained using an elegant strategy. Retrograde tracing and electrophysiological recordings showed that the ventrotemporal retina contains nine types of ipsilateral RGCs among the 40 or so known types of RGCs previously characterized in mice ( Johnson et al. 2021). These include several types of intrinsically photoreceptive RGCs. Elegant whole-cell patch-clamp recordings further demonstrated that prey capture relies on no more than five types of ipsilateral RGCs.

In contrast to species with robust binocular vision (e.g., cats, nonhuman primates, and humans) in which ipsilateral and contralateral inputs are segregated in vertical ocular dominance columns in the cortex, mice possess a small binocular area with neurons responding to visual stimuli from both eyes and a larger monocular area in the visual cortex (Datwani et al. 2009). Recent studies defend the existence of true ocular dominance columns in rodents (Goltstein et al. 2023, Ohki et al. 2005, Ringach et al. 2016), but to what extent they are necessary for binocular visual processing has yet to be determined.

The importance of ipsilateral RGCs in stereopsis and predation cannot be discounted, but such properties have also been characterized in vertebrate species with complete chiasm decussation such as zebrafish, owls, and toads through other circuits (Read 2023). In zebrafish, intertectal commissural connections allow brain nuclei processing visual information to receive and compare visual inputs from both eyes despite having no ipsilateral axons (Gebhardt et al. 2019). Interestingly, stereopsis also exists in invertebrate predators such as the praying mantis and the cuttlefish (Feord et al. 2020). The neural circuits underlying stereopsis in these species are unknown, but they are probably wired quite differently than in vertebrates.

## CONCLUSIONS AND FUTURE DIRECTIONS

The last several decades have witnessed great progress in understanding the guidance of RGC axons from the eye to the brain as the circuit for binocular vision is formed and the molecular mechanisms that mediate decussation at the optic chiasm, implement coherence of growth in the optic tract, and enable target entry and matching. With methodological advances in identifying genes for transcription factors, the progression of precursor cells to defined cellular populations and the signaling pathways these transcription factors control have been elucidated. Advances in imaging and techniques for labeling and observing in three dimensions in intact brain have led to novel findings on the evolution of the binocular circuit. And the use of the mouse as a genetically tractable model has led to new insights on binocular visual function and the retinal cells responsible for it.

Many questions remain. At the molecular level, how does the multitude of guidance factors (e.g., semaphorins, VEGF, Shh, and Wnt signaling) interact to produce essentially a binary response in axon navigation at the optic chiasm? When and how does the cascade of transcription factor expression designate the separation of ipsi- and contralateral RGC identity. Ipsi- and contralateral RGC axons segregate in the optic tract, quite far from their targets. Is pretarget organization of ipsi- versus contralateral axons in the optic tract a prerequisite for proper targeting? What molecules implement this segregation, and are they important for targeting? What are the molecules involved in activity-dependent segregation?

At the circuit/functional level, although a binocular circuit exists in lower vertebrates, especially ancient fishes, and even in invertebrates, this circuit does not seem to correlate with eye position, terrestrial habitats, or transcriptional encoding. It is possible that the binocular circuit may not be as related to stereopsis as previously thought. What is the molecular signaling for the ipsilateral fate in the absence of Zic2 in nonmammalian species? Do mechanisms similar to those shown in mice hold for the human eye-brain circuit, where nearly half of the RGCs project ipsilaterally?

With leaps in technical prowess in the field at large, answers to some of these questions will surely come rapidly.
